# Supplementary material for: The Role of Late Presenters in HIV-1 Transmission Clusters in Europe
Source: Viruses. 2023 Dec 13;15(12):2418. doi: 10.3390/v15122418 (PMC10746990; doi:10.3390/v15122418)
Supplement: Supplementary file 1 [file viruses-15-02418-s001.zip › viruses-2720892-supplementary.pdf]

Table Supplementary S1. Patients socio-demographic and clinical characteristics

Patient Characteristics	Total	Subtype A	Subtype B	Subtype G	p-value
**Total**	2679 (100)	168 (6·3)	2437 (91·0)	74 (2·8)	
**Sex, n (%)**	2655 (99·1)	167 (6·3)	2414 (90·9)	74 (2·8)	<0.001
Male	2262 (85·2)	95 (4·2)	2136 (94·4)	31 (1·4)	
Female	393 (14·8)	72 (18·3)	278 (70·7)	43 (10·9)	
**Median age at resistance teste in years IQR, n (%)**	2660 (99·3)	167 (6·3)	2419 (90·9)	74 (2·8)	0.001
	39·0 (31·0-47·0)	39·0 (31·0-48·0)	39·0 (31·0-47·0)	34·0 (28·0-41·0)	
≤ 18	8 (0·3)	1 (12·5)	7 (87·5)	0 (0)	<0.021
19-30	605 (22·7)	40 (6·6)	537 (88·8)	28 (4·6)	
31-55	1809 (68·0)	105 (5·8)	1661 (91·8)	43 (2·4)	
≥ 56	238 (8·9)	21 (8·8)	214 (89·9)	3 (1·3)	
**Transmission Route, n (%)**	1978 (73·8)	120 (6·1)	1806 (91·3)	52 (2·6)	<0.001
Heterosexual	644 (32·6)	61 (9·5)	547 (84·9)	36 (5·6)	
MSM	1189 (60·1)	23 (1·9)	1160 (97·6)	6 (0·5)	
IDU	105 (5·3)	24 (22·9)	80 (76·2)	1 (1·0)	
Other	40 (2·0)	12 (30·0)	19 (47·5)	9 (22·5)	
**Region of origin, n (%)**	2216 (82·7)	150 (6·8)	2000 (90·3)	66 (3·0)	<0.001
Western Europe	1883 (85·0)	80 (4·2)	1785 (94·8)	18 (1·0)	
Eastern Europe	121 (5·5)	40 (33·1)	78 (64·5)	3 (2·5)	
Africa	98 (4·4)	24 (24·5)	30 (30·6)	44 (44·9)	
South America	66 (3·0)	0 (0)	65 (98·5)	1 (1·5)	
Other	48 (2·2)	6 (12·5)	42 (87·5)	0 (0)	
**Migration Status**	2216 (82·7)	150 (6·8)	2000 (90·3)	66 (3·0)	<0.001
Migrant	407 (18·4)	80 (19·7)	275 (67·6)	52 (12·8)	
Native	1809 (81·6)	70 (3·9)	1725 (95·4)	14 (0·8)	
**Clusters**	2679 (100)	168 (6·3)	2437 (91·0)	74 (2·8)	<0.001
In Clusters	1116 (41·7)	39 (3·5)	1060 (95·0)	17 (1·5)	
Out-of-Clusters	1563 (58·3)	129 (8·3)	1377 (88·1)	57 (3·6)	
**Recentness of infection**	2679 (100)	168 (6·3)	2437 (91·0)	74 (2·8)	<0.001
Recent	1156 (43·2)	51 (4·4)	1081 (93·5)	24 (2·1)	
Chronic	1523 (56·8)	117 (7·7)	1356 (89·0)	50 (3·3)	
**TDR**	2679 (58·4)	168 (6·3)	2437 (91·0)	74 (2·8)	0.040
Yes	282 (10·5)	9 (3·2)	268 (95·0)	5 (1·8)	
No	2397 (89·5)	159 (6·6)	2169 (90·5)	69 (2·9)	
**Median CD4 count at diagnosis (cells/mL) IQR, n (%)**	2609 (97·4)	160 (6·1)	2377 (91·1)	72 (2·8)	0.442
	320·0 (134·0-506·5)	288·5 (129·25-468·25)	321·0 (131·5-511·5)	318·0 (168·75-430·75)	
LP	1426 (54·7)	98 (6·9)	1287 (90·3)	41 (2·9)	0.201
NLP	1183 (45·3)	62 (5·2)	1090 (92·1)	31 (2·6)	
**Viral Load at diagnosis (log_{10} copies/mL) IQR, n (%)**	2630 (57·3)	165 (6·3)	2392 (91·0)	73 (2·8)	0.071
	4·7 (4·3-5·4)	4·5 (3·9-5·2)	4·8 (4·0-5·4)	4·5 (3·8-5·3)	
≤ 4.0	641 (24·4)	50 (7·8)	567 (88·5)	24 (3·7)	0.103
4.1-5.0	939 (35·7)	54 (5·8)	858 (91·4)	27 (2·9)	
≥ 5.1	1050 (39·9)	61 (5·8)	967 (92·1)	22 (2·1)	

Table Supplementary S2. Unadjusted analysis for determinants associated with belonging to a transmission cluster according to Subtype A, B and G

		Subtype A		Subtype B		Subtype G	
**In clusters/Out-of-clusters**		**Unadjusted Model**		**Unadjusted Model**		**Unadjusted Model**	
		**uOR (95%CI)**	**p-value**	**uOR (95%CI)**	**p-value**	**uOR (95% CI)**	**p-value**
**Sex**	**Female**	Ref	Ref	Ref	Ref	Ref	Ref
	**Male**	4·72 (1·94-11·47)	**0·001**	1·32 (1·02-1·70)	**0·037**	1·79 (0·60-5·33)	0.296
**Age at resistance test**	**<18**	-	1.000	1·84 (0·40-8·41)	0·433	-	0.999
	**19-30**	1·07 (0·34-3·43)	0·907	1·23 (0·89-1·69)	0·209	-	0.999
	**31-55**	0·59 (0·20-1·71)	0·329	1·01 (0·76-1·35)	0·940	-	0.999
	**>56**	Ref	Ref	Ref	Ref	Ref	Ref
**Transmission Route**	**Heterosexual**	Ref	Ref	Ref	Ref	Ref	Ref
	**MSM**	4·46 (1·59-12·52)	**0·005**	1·52 (1·23-1·87)	**<0·001**	5·00 (0·81-31·00)	0.084
	**IDU**	0·58 (0·15-2·28)	0·439	1·43 (0·89-2·30)	0·138	-	1.000
	**Other**	0·37 (0·04-3·16)	0·365	1·57 (0·63-3·94)	0·332	-	0.999
**Region of Origin**	**Western Europe**	Ref	Ref	Ref	Ref	Ref	Ref
	**Eastern Europe**	0·44 (0·17-1·13)	0·088	0·92 (0·58-1·46)	0·720	0·79 (0·06-10·38)	0.855
	**Africa**	0·19 (0·04-0·86)	**0·032**	0·63 (0·29-1·35)	0·231	0·20 (0·05-0·76)	**0.018**
	**South America**	-	-	0·70 (0·37-1·32)	0·267	-	1.000
	**Other**	2·08 (0·39-11·00)	0·390	0·78 (0·47-1·30)	0·347	-	-
**Migration Status**	**Migrant**	Ref	Ref	Ref	Ref	Ref	Ref
	**Native**	2·86 (1·33-6·18)	**0·007**	1·15 (0·89-1·50)	0·277	4·13 (1·13-15·12)	**0.033**
**Recentness of Infection**	**Chronic**	Ref	Ref	Ref	Ref	Ref	Ref
	**Recent**	3·33 (1.58-7.02)	**0·002**	2·30 (1·95-2·71)	**<0·001**	4·39 (1·41-13·70)	**0.011**
**TDR**	**Yes**	Ref	Ref	Ref	Ref	Ref	Ref
	**No**	1·06 (0·21-5·33)	0·942	1·16 (0·90-1·50)	0·263	1·21 (0·13-11·59)	0.870
**LP/NLP**	**LP**	Ref	Ref	Ref	Ref	1	1
	**NLP**	2·44 (1·16-5·12)	**0·018**	1·34 (1·14-1·58)	**<0·001**	1·44 (0·47-4·38)	0.526
**Viral load groups**	**<4.0**	Ref	Ref	Ref	Ref	1	1
	**4.1-5.0**	1·95 (0·71-5·37)	0·197	1·22 (0·98-1·51)	0·069	0·69 (0·20-2·46)	0.571
	**>5.1**	2·57 (0·98-6·78)	0·056	1·05 (0·85-1·29)	0·665	0·38 (0·09-1·72)	0.211
